# Numerical Investigation of Composite Behavior and Strength of Rectangular Concrete-Filled Cold-Formed Steel Tubular Stub Columns

**DOI:** 10.3390/ma14206221

**Published:** 2021-10-19

**Authors:** Liping Wang, Yanan An, Faxing Ding, Yachuan Kuang, Qing Ma, Sui Tan, Weizhen Zhang, Pengzhou Zhao, Enhui Ren

**Affiliations:** 1 School of Civil Engineering, Central South University, Changsha 410075, China; wlp2016@csu.edu.cn (L.W.); anyananlg@163.com (Y.A.); dinfaxin@csu.edu.cn (F.D.); maqing18707492932@163.com (Q.M.); 2 Engineering Technology Research Center for Prefabricated Construction Industrialization of Hunan Province, Changsha 410075, China; 3 National Engineering Laboratory for High Speed Railway Construction, Changsha 410075, China; sunnytansui@csu.edu.cn; 4 Hunan Zhongda Design Institute Co., Ltd., Changsha 410075, China; linquanzhang@yeah.net; 5 Hunan Zhongtian Hangxiao Steel Structure Technology Co., Ltd., Changsha 410075, China; pengzhouzhao@163.com; 6 Guangdong Architectural Design & Research Institute Co., Ltd., Guangzhou 510170, China; reh@gdadri.com

**Keywords:** cold-formed steel, composite behavior, concrete-filled steel tube, confinement coefficient, ultimate bearing capacity

## Abstract

The objective of this study was to investigate the composite behavior of rectangular concrete-filled cold-formed steel (CFS) tubular stub columns under axial compression. A fine finite 3D solid element model of rectangular concrete-filled cold-formed steel tubular stub column was established by ABAQUS, which utilized a constitutive model of cold-formed steel considering the cold-forming effect and a triaxial plastic-damage constitutive model of the infilled concrete. Good agreement was achieved and the average discrepancy between the experimental and FE results was less than 5%. Based on the verified models, a further parametric analysis was carried out to reveal the influence of various factors on the strength and behavior of the concrete-filled rectangular cold-formed steel tubular stub columns. The factors included constitutive models adopted for cold-formed steel, length over width ratio of the rectangular section, wall-thickness and width, and concrete strength and yield strength of the cold-formed steel. A total of 144 FE models were analyzed. The stress nephogram was reasonably simplified in accordance with the limit state and a theoretical formula considering confinement coefficient was proposed to estimate the ultimate bearing capacity of concrete-filled rectangular cold-formed steel tubular stub columns using the superposition method. The calculated results showed satisfactory agreement with both the experimental and FE results, which proved the validity and accuracy of the formula proposed in this paper. In the proposed formula, the confinement coefficient of square concrete-filled cold-formed steel tubular stub columns is larger than that of hot-rolled steel counterparts but smaller than that of the stainless steel counterparts.

## 1. Introduction

Concrete-filled steel tube (CFST) is a preferable combination of steel and concrete materials, which can make the most use of the compressive strength of concrete and the tensile strength of steel. Apart from maintaining the advantages of CFST, concrete-filled cold-formed steel tube (CFCFST) has the following characteristics compared to the welded steel tube: higher welding quality can be guaranteed and less construction time is required due to fewer weld seams, and less residual stress and residual deformation results from the welding process. Therefore, CFCFST members have been increasingly used in building construction.

Until now, the research and design theories on CFST columns have achieved good development [1,2,3,4,5,6,7] and relevant outcomes have been included in design codes around the world. However, investigations for CFCFST stub columns are still limited. A series of experimental studies and finite element analyses have been accomplished and the mechanical performance of CFCFST stub columns under axial compression were investigated. Furthermore, the formulas for calculating the bearing capacity of stub columns under axial compression were put forward [8,9,10,11,12]. Zhu et al. [13] explored the influence of stiffeners on the mechanical performance of square CFCFST stub columns through experimental studies and the calculated results using different codes were compared with the test results, which showed that the calculated results in the current codes are conservative. A series of tests have been carried out and the mechanical performance of rectangular CFCFST stub columns and elliptical CFCFST stub columns investigated [14,15], indicating that the predicted results using the design method in EC4 are reliable. The mechanical performance of CFCFST stub columns under different axial loading modes were also discussed through experimental studies and FE analysis [16,17]. Moreover, the hysteretic behavior of CFCFST columns with rectangular and circular cross-sections have been investigated and discussed in several studies [18,19].

The research group of the authors has carried out a series of experimental and FE analyses on CFST stub columns under axial compression [20,21,22,23,24]. A unified design formula was proposed to predict the bearing capacity of rectangular CFST stub columns with both hot-rolled steel tubes and stainless steel tubes, based on their own results and the existing experimental data pool. At the same time, a comparative study on the mechanical performance of concrete-filled square hot-rolled steel tubular and concrete-filled square stainless steel tubular stub columns under axial loading was carried out. It was found that the confinement coefficient between the outer steel tube and core concrete in the design formula is 1.2 for hot-rolled steel tube and 1.4 for the square stainless steel tube, due to the larger strengthening modulus of stainless steel.

To sum up, the mechanical performance of rectangular CFCFST stub columns has gained increasing research interest over recent years. However, the influence of different constitutive models of cold-formed steel on the composite behavior have not yet been thoroughly investigated. Previous numerical simulations mainly investigated the factors having influence on the mechanical behavior of CFCFST stub columns under axial load. The composite behaviors of rectangular CFCFST stub columns were rarely discussed and the different confinement coefficients among square CFCFST stub columns, concrete-filled square hot-rolled steel tubular stub columns, and concrete-filled square stainless steel tubular stub columns have not yet been discussed.

Therefore, this study aimed to identify the composite behaviors between cold-formed steel tubes and core concrete and was carried out as follows: (1) A fine finite 3D solid element model of rectangular CFCFST stub column was established using ABAQUS by adopting a constitutive model of cold-formed steel considering the cold-forming characteristics and a triaxial plastic-damage constitutive model of concrete under axial compression, and non-linear finite element analysis was carried out to simulate the whole loading process of CFCFST stub columns. (2) Based on the numerical model that was verified against experimental results, a parametric study was performed and the composite behavior of cold-formed steel tube on concrete with different sectional sizes, steel ratios, steel yield strengths, and concrete strengths was investigated. The mechanical properties of rectangular CFCFST stub columns under axial compression based on different constitutive models of cold-formed steel were also investigated. (3) According to the results of parametric study and the equilibrium theory at limit state, a practical design formula considering the confinement coefficient was proposed to predict the ultimate bearing capacity of the rectangular CFCFST stub columns under axial compression.

## 2. Finite Element Modeling and Analysis

### 2.1. Finite Element Models

#### 2.1.1. Element Types and Mesh Generation

**Figure 1:**
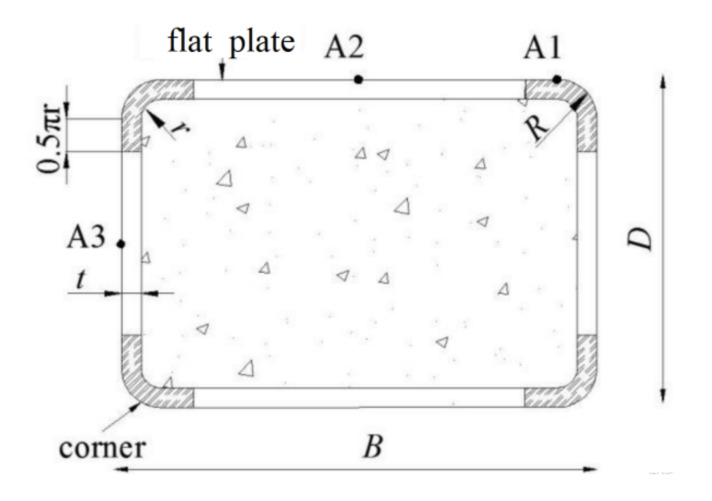
Cross-section configuration.

**Figure 2:**
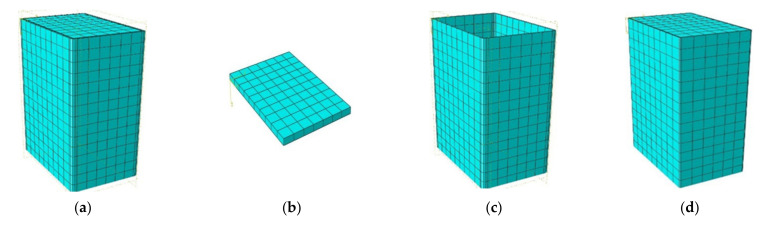
Mesh generation of the FE models: (**a**) FE model, (**b**) loading plate, (**c**) cold-formed steel tube, and (**d**) core concrete.

Wei et al. [25] revealed that stress–strain curves may be considerably influenced by the corner radius for rectangular concrete-filled fiber reinforced polymer (FRP)–steel composite tube columns and the ultimate strength increased with increasing corner radius. Ouyang et al. [26] found that increasing the corner radius would produce better confinement at post-peak stage and thus would improve the post-peak behavior of square CFST columns by FE analysis. Therefore, the dimensions of the cold-formed steel tube in the finite element study were selected in accordance with code [27]. The outer diameter *R* = 2*t* and the inner diameter *r* = *t* at the corner of the section were fixed. The wall-thickness, long-side, and short-side of the cold-formed steel tube were *t*, *B* and *D*, respectively. The cross-section configuration of the FE models is shown in Figure 1. The FE models of CFCFST stub columns were established using ABAQUS, which consisted of three parts—the cold-formed steel tube, the core concrete, and the loading plate. In the numerical models, the 8-node reduced integral format 3D solid element (C3D8R) was applied to model the cold-formed steel tube, core concrete and loading plate for all specimens. The structured meshing technique was adopted as shown in Figure 2.

Surface-to-surface contact was adopted for the interaction between cold-formed steel tube and core concrete. Limited glide was employed in the sliding formulation, and the discretization method was surface-to-surface. The normal behavior was set to “hard” contact, meanwhile tangential behavior was defined in the contact property to simulate the interfacial bond—glide relationship between cold-formed steel tube and core concrete. The penalty function was utilized to the friction formula for the tangential behavior. The friction coefficient of 0.5 has been successfully used for simulating hot-rolled steel concrete-filled tubular columns, and the bond behavior has very minor influence on the performance of stub columns of different steel [21]. Therefore, the friction coefficient of 0.5 was used in this study. The tie constraint was chosen for the cold-formed steel tube, core concrete, and loading plate so that the load could be applied to the all specimens in the whole loading process. A rigid body was used to simulate the loading plate in which the elasticity modulus was taken as 1.0 × 10^12^ MPa and the Poisson’s ratio was set as 1.0 × 10^−7^.

Full-scale FE models were established to investigate the mechanical behavior of CFCFST stub columns under axial compression. Loading was applied in a displacement control mode on top of the stub column to simulate the axial loading condition. In addition, the incremental iterative method was used to consider structural nonlinearities during the analysis.

#### 2.1.2. Material Constitutive Models

A triaxial plastic-damage constitutive model of concrete under axial compression proposed by the authors was adopted in the model [28].

(1) $$y=\left\{\begin{array}{ll} \frac{A_{1}x+(B_{1}-1)x^{2}}{1+(A_{1}-2)x+B_{1}x^{2}} & x\leq 1\\ \frac{x}{\alpha_{1}{\left(x-1\right)}^{2}+x} & x>1 \end{array}\right.$$

where *y* = *σ*/*f*_c_, *x* = *ε*/*ε*_c_. *f*_c_(=0.4*f*_cu_^7/6^), and *f*_cu_ are the uniaxial compressive concrete strength and cubic compressive concrete strength, respectively. *ε*_c_ is the strain at peak stress and expresses 383 *f*_cu_^7/18^ × 10^−6^. *A*_1_ is the ratio of the initial tangent modulus to the secant modulus at peak stress and expresses to 9.1*f_cu_*^−4/9^; *B*_1_ = 1.6(*A*_1_ − 1)^2^ is a parameter that controls the decrease in the elastic modulus along the ascending branch of the axial stress–strain relationship. For confined concrete structures, parameter *α*_1_ can be taken as 0.15.

Poisson’s ratio is taken as 0.2 in the elastic stage of concrete. The plastic-damage constitutive model was defined in ABAQUS—the eccentricity is 0.1, the ratio of initial equibiaxial compressive yield stress to initial uniaxial compressive yield stress is 1.225, the ratio of the second stress invariant on the tensile meridian to that on the compressive meridian is 0.6667, the viscosity parameter is 0.0005, and the dilation angle is 40°. The concrete model has been successfully validated for CFST stub columns with various sections [20].

The four-fold line constitutive model of cold-formed steel proposed by Abdel-Rahman et al. [29] was adopted in the finite element analysis for test verification.

(2) $$\sigma =\left\{\begin{array}{ll} E_{s}\varepsilon & \varepsilon \leq \varepsilon_{e}\\ f_{p}+E_{s1}(\varepsilon -\varepsilon_{e})\; & \varepsilon_{e}<\varepsilon \leq \varepsilon_{e1}\\ f_{\mathrm{sm}}+E_{s2}(\varepsilon -\varepsilon_{e1})\; & \varepsilon_{e1}<\varepsilon \leq \varepsilon_{e2}\\ \;f_{s}+E_{s3}(\varepsilon -\varepsilon_{e2})\; & \varepsilon >\varepsilon_{e2} \end{array}\right.$$

where *f*_s_ is the yield strength of cold-formed steel, *E*_s_ (=2.06 × 10^5^ MPa) is the elastic modulus, *E*_s1_ (=0.5 *E*_s_), *E*_s2_ (=0.1 *E*_s_) and *E*_s3_ (=0.005 *E*_s_) are the slopes of second to fourth part of the four-fold line model, and *f*_p_ = 0.75*f*_s_, *f*_sm_ = 0.875*f*_s_, *ε*_e_ = 0.75*f*_s_/*E*_s_, *ε*_e1_ = *ε*_e_ + 0.125*f*_s_/*E*_s1_, and *ε*_e2_ = *ε*_e1_ + 0.125 *f*_s_/*E*_s2_.

There is strengthening effect at the corner and the yield strength is improved to be:

(3) $$f_{\mathrm{sy}}=[0.6h/{\left(r/t\right)}^{m}+0.4]f_{s}$$

where *f*_sy_ is the yield strength of cold-formed steel tube at the corner, *h* = 3.69(*f*_u_/*f*_s_) − 0.819(*f*_u_/*f*_s_)^2^ − 1.79, and *m* = 0.192(*f*_u_/*f*_s_) − 0.068. *f*_u_ is the ultimate strength of cold-formed steel.

### 2.2. Model Validation

**Figure 3:**
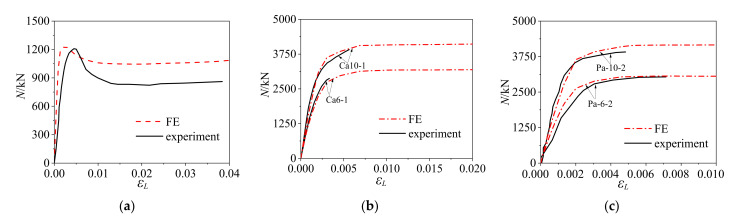
Comparison of FE and experimental load–strain curves: (**a**) CFT-SC [8], (**b**) Ca6-1 and Ca10-1 [9], and (**c**) Pa-6-2 and Pa-10-2 [13].

In this paper, the FE models of rectangular CFCFST stub columns under axial compression were established using the same modelling methods as reported by Ding et al. [21]. The four-fold line constitutive model of cold-formed steel presented in the previous section were adopted in the model. The obtained finite element results were verified against the experimental results reported by Tao et al. [8], Li [10], Zhang [9], Zhu et al. [13], Ferhoune [14], and Qu et al. [17]. The ultimate bearing capacity of FE results (*N*_u,FE_) were compared with the experimental results (*N*_u,e_) in Table 1. It was shown that the average ratio of *N*_u,e_ to *N*_u,FE_ was 0.97 with the corresponding dispersion coefficient of 0.07. From the above comparison, it can be seen that generally good agreement was achieved between the FE and test results, although the FE results were slightly larger which may have been due to the fact that the imperfections of the experimental specimens were not fully reflected in the models. The typical load–strain curves of FE results and experimental results [8,9,13] were compared as shown in Figure 3. It can be seen that the ultimate bearing capacity obtained from FE results and the available test results were in good agreement. The measured deformation in the test was commonly larger than that obtained from FE analysis, resulting in the stiffness of the specimens in the elastic stage being smaller. This may have been due to the fact that there were initial gaps between the contacting plates in the test setup. In adddition, local buckling and steel tube tearing failure occurred with the specimens after the peak value, and then the bearing capacity declined rapidly. The FE method and constitutive models have limitations; they cannot simulate the crack of the specimen and the crushing of core concrete after the peak value, leading to the higher curve of the FE result compared with experimental result. However, this difference would not affect the ultimate capacity of the stub columns. Both the FE results and the experimental studies had the same trends—the bearing capacity was positively correlated with wall-thickness, concrete strength, and yield strength of cold-formed steel. Therefore, the FE models can be used to carry out further parametric study of the CFCFST stub columns beyond the range of the test specimens.

### 2.3. Parametric Study

**Figure 4:**
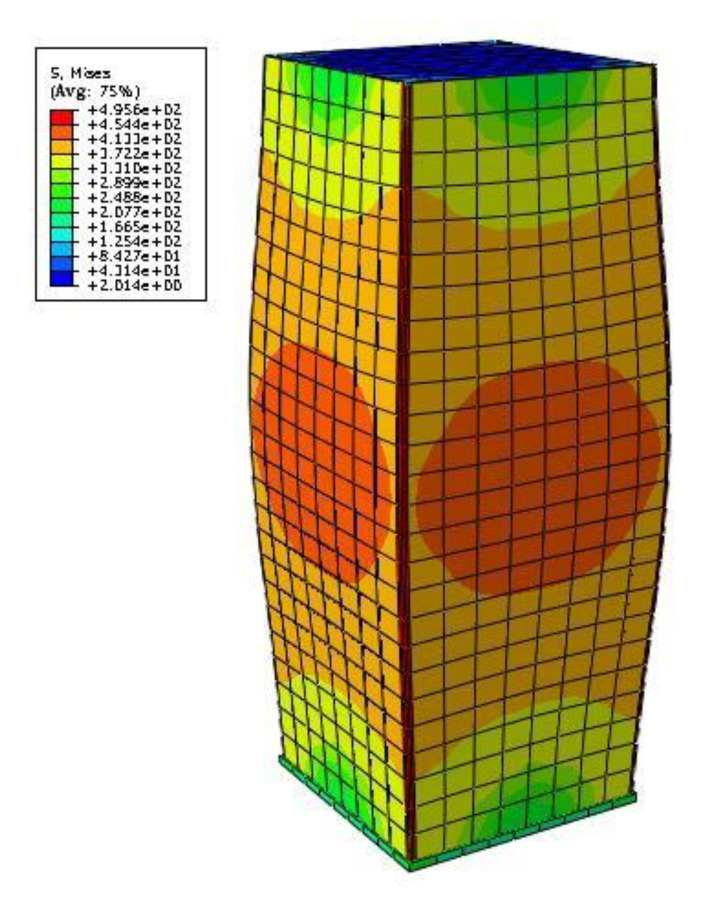
FE stress nephogram of CFCFST stub column at ultimate limit state.

In the parametric study, both square and rectangular cold-formed steel sections with different dimensions were investigated. *D* = 300 mm, 400 mm, or 500 mm was taken when *B*/*D* = 1 (square sections); *D* = 500 mm was used when *B*/*D* = 1.5, 2, or 3 (rectangular sections) in the FE models. The steel ratio *ρ*_s_ ranged from 0.02 to 0.08 and the wall-thickness of steel tube ranged from 2 mm to 15 mm. The yield strength *f*_s_ of cold-formed steel was taken as 235 MPa, 345 MPa, or 450 MPa; the cubic compressive concrete strength *f*_cu_ was taken as 40 MPa, 60 MPa, 80Mpa, or 100 MPa. Considering the practical engineering use, cold-formed steel of *f*_s_ = 235 MPa was paired with the concrete grade of C40 or C60; *f*_s_ = 345 MPa was paired with C60 or C80; and *f*_s_ = 450 MPa was paired with C80 or C100. A total of 144 FE models were analyzed with consideration of all the above parameters. Figure 4 is the typical FE stress nephogram of square CFCFST stub column at ultimate limit state.

#### 2.3.1. Constitutive Models of Cold-Formed Steel

The constitutive relations of cold-formed steel reflect the material characteristics and cold-forming effect [30,31,32]. At present, the constitutive models of cold-formed steel tube used for FE analysis mainly include a four-fold line model, an ideal elastoplastic model, and a BKIN model, shown as Equations (2)–(5).

(4) $$\sigma =\left\{\begin{array}{ll} E\varepsilon & \varepsilon \leq \varepsilon_{y}\\ f_{y} & \varepsilon >\varepsilon_{y} \end{array}\right.$$

(5) $$\sigma =\left\{\begin{array}{ll} E\varepsilon & \varepsilon \leq \varepsilon_{y}\\ f_{y}+E_{t}(\varepsilon -\varepsilon_{y})\; & \varepsilon >\varepsilon_{y} \end{array}\right.$$

where, *f*_y_ and *ε*_y_ are the yield strength and yield strain of steel, respectively, *E* (=2.06 × 10^5^ MPa) is the elastic modulus of steel, and *E*_t_ (=0.01 *E*) is the tangent modulus in the plastic strengthening stage.

The four-fold line model and ideal elastoplastic model as well as BKIN model were considered in the comparative study in order to identify the influence of constitutive models of cold-formed steel tube. Taking the model of *B/D* = 1, *B* = 500 mm, *t* = 4 mm, and *f*_s_ = 345 MPa paired with C60 concrete as an example, the composite behavior between steel tube and concrete were compared and analyzed.

**Figure 5:**
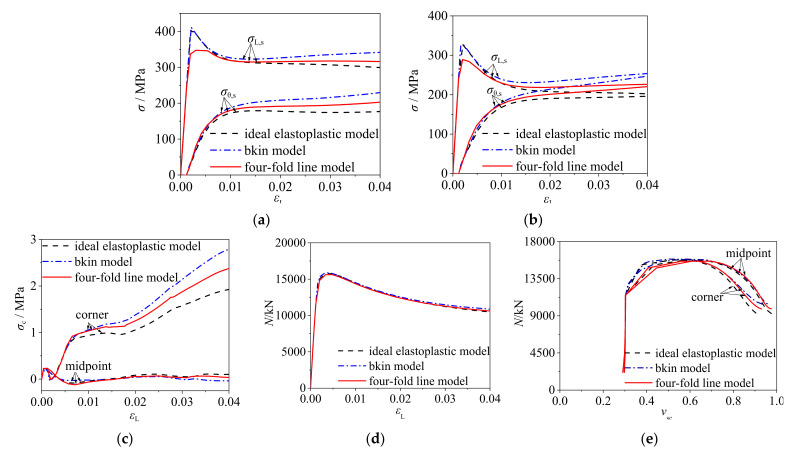
Comparison of typical curves for CFCFST adopting different constitutive models of cold-formed steel: (**a**) stress–strain curves of steel tubes at point A1, (**b**)stress–strain curves of steel tubes at point A2, (**c**) radial stress–strain curves of core concrete, (**d**) load–strain curves, and (**e**) load–strain ratio (*N*-*ν*_sc_) curves of steel tubes.

Figure 5a,b present the axial stress (*σ*_L,s_) and transverse stress (*σ*_θ,s_) curves of cold-formed steel at endpoint (A1 in Figure 1) and midpoint (A2 in Figure 1). Ding et al. [20] proposed a method for evaluating the degree of composite behavior between the steel tube and core concrete by analyzing the relation of axial stress and transverse stress curves. If the axial stress curve intersects with the transverse stress curve, it demonstrates that the composite action is strong. Further, if the intersection occurs earlier, the confinement efficiency is higher, and vice versa. At the initial loading, the transverse stresses at the point A1 and point A2 of steel tubes based on three different constitutive models were basically the same, while the axial stresses of steel tubes based on ideal elastoplastic and BKIN models were higher and there were sharp angles. At this time, the steel tube was mainly participating in axial compression, and the transverse stress was small. By comparing the results based on the three constitutive models of cold-formed steel, it is found that: (1) The stress–strain curves based on the ideal elastoplastic and BKIN models ended the linear elastic stage when the steel tube reached the yield strength, but the curve based on four-fold line model had a transitional stage between the proportional limit and the yield limit, and the curve based on four-fold line model entered the nonlinear stage earlier. Therefore, there was no obvious peak angle in the curve based on four-fold line model. (2) The axial and transverse stress of the steel tube based on four-fold line model at the endpoint (Figure 5a) and midpoint (Figure 5b) was larger than that based on the ideal elastoplastic model but smaller than that based on the BKIN model, and the amplitude of variation of stress was basically the same among the three groups of curves. Different models of steel material have influences on the composite behavior, and the four-fold line model closest to the stress–strain curve of cold-formed steel was recommended in this study.

Figure 5c shows the radial stress–strain curves of core concrete in CFCFST stub columns and it indicates the lateral compressive stress provided by the steel tube on the core concrete. As shown in Figure 5c, the radial stress of the core concrete based on the four-fold line model at the corner was greater than that with ideal elastoplastic model but smaller than that with BKIN model, while the radial stress based on three models was close and small at the midpoint. This suggests that the confinement effect of cold-formed steel tube on core concrete based on the four-fold line model was greater than that with ideal elastoplastic model, but weaker than that with BKIN model. The composite behavior for all specimens at endpoint (corner) was greater than that at midpoint.

As illustrated in Figure 5d, the ultimate bearing capacities of CFCFST stub columns based on the three constitutive models were close. It indicates that the three constitutive models of steel tube had little effect on the overall bearing capacity of CFCFST stub columns. Figure 5e presents the load–strain ratio curves of CFCFST stub columns. *ν*_sc_ is the ratio of transverse strain to axial strain, reflecting the confinement effect of steel tube on core concrete. The larger *ν*_sc_ is, the stronger the confinement effect. At the initial loading, the *ν*_sc_ did not change much but the load rose sharply, which indicates that the steel tube mainly participated in axial compression and had little constraint on the core concrete. At about 70% of the ultimate load, the *ν*_sc_ based on the four-fold line model appeared to be at the critical point and increased relatively quickly, indicating that the steel tube yields and the confinement effect on core concrete developed earlier. At the ultimate load, the intersection point of *N*-*ν*_sc_ appeared and the confinement effect of steel tube on core concrete based on three constitutive models was close. After the ultimate load, taking the *ν*_sc_ based on the four-fold line model as a benchmark, the *ν*_sc_ based on BKIN model was larger while *ν*_sc_ based on ideal elastoplastic model was smaller under the same load. The results suggest that the composite behavior of steel tube on concrete based on four-fold line model was greater than that on ideal elastoplastic model but weaker than that on BKIN model. In the whole loading process, the *ν*_sc_ was less than 1. This indicates that there was friction between steel tube and core concrete, so that the steel tube mainly participated in axial compression. To sum up, different models have influence on the composite behavior but a very minor influence on the ultimate load capacity. The four-fold line model was recommended in this study because it is closest to the stress–strain curve of cold-formed steel.

#### 2.3.2. Length over Width Ratio (*B*/*D*) of Rectangular Section

**Figure 6:**
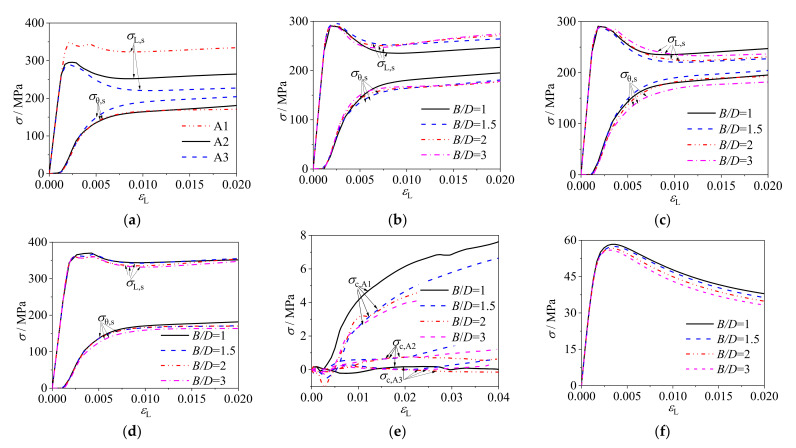
Stress–strain curves of rectangular CFCFST stub columns with different *B*/*D*: (**a**) stress–strain curves of steel tubes at different measuring points, (**b**) stress–strain curves of steel tubes at long-side midpoint A2, (**c**) stress–strain curves of steel tubes at short-side midpoint A3, (**d**) stress–strain curves of steel tubes at endpoint A1, (**e**) radial stress–strain curves of core concrete, and (**f**) average axial stress of core concrete.

The FE analysis adopted the models of *f*_s_ = 345 MPa, C60, and *ρ*_s_ = 0.05 with different *B*/*D* ratios. The representative points were selected at endpoint A1, long-side midpoint A2 and short-side midpoint A3 as presented in Figure 1. The axial and transverse stress–strain curves of steel tube and the axial and radial stress–strain curves of core concrete are shown in Figure 6. Comparing the relation curves, it is found that:

(1) As shown in Figure 6a–d, the distance between the *σ*_L,s_-*ε*_L_ curve and the *σ*_θ,s_-*ε*_L_ curve at point A2 is farther than that at point A3, which indicates that the confinement efficiency on the short-side was higher than that on the long-side of the rectangular column. With the increase of *B*/*D*, the distance between the *σ*_L,s_ − *ε*_L_ curve and the *σ*_θ,s_ − *ε*_L_ curve increased and the overall confinement efficiency decreased.

(2) As shown in Figure 6e–f, the radial stress of core concrete at endpoint A1 was far greater than that at midpoints (A2 and A3) and it reflects that the confinement effect at endpoint was larger. With the increase of *B*/*D*, the radial stress decreased at endpoint, while the changes at midpoint were not obvious. The axial stress of core concrete decreases with the increase of *B*/*D*. It can be regarded that the confinement effect of cold-formed steel tube on core concrete decreases with the increase of *B*/*D.*

#### 2.3.3. Wall-Thickness and Width

**Figure 7:**
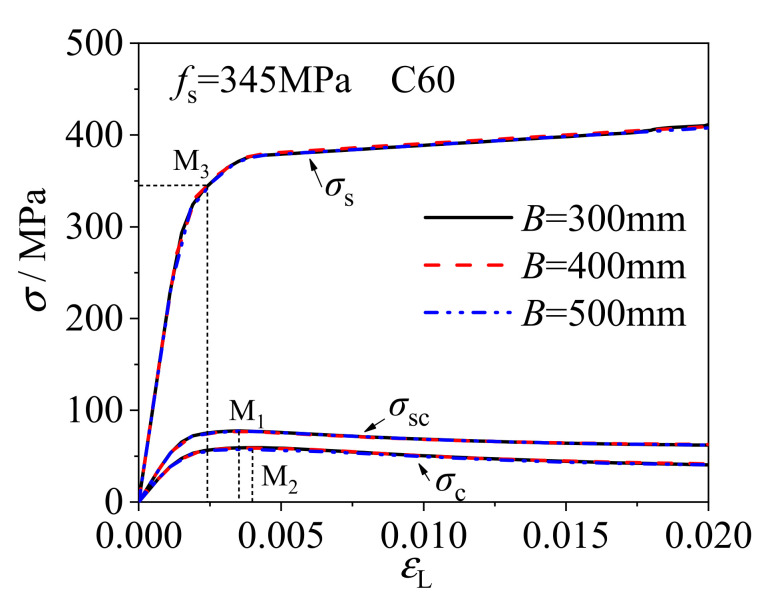
The influence of *B* on the mechanical performance of square CFCFST stub columns (*B*/*t* = constant).

Taking the square CFCFST stub columns of steel ratio *ρ*_s_ = 0.08 as an example, the stress of steel tube and core concrete is obtained from the numerical analysis. The overall average stress of CFCFST stub column is *σ*_sc_ = *N*/*A*_sc_ (*A*_sc_ = *A*_c_+*A*_s_). The equivalent average stress of steel tube *σ*_s_ is determined by the Von Mises yield criterion. Figure 7 shows the comparison of stress–strain curves of overall average stress (*σ*_sc_), average stress of concrete (*σ*_c_), and equivalent average stress of steel tube (*σ*_s_) under three cross-section sizes of *B* = 300 mm, 400 mm, and 500 mm. The corresponding thicknesses of three sections were 6mm, 8mm, and 10 mm, respectively, resulting the same *B*/*t* ratio of 50 for three sections. It can be seen that the three curves are almost identical and there was no significant effect on CFCFST stub columns under different cross-section sizes with the same *B*/*t* ratio. The yield strength of steel tube (M_3_) appeared before the overall average stress reached the maximum (M_1_ is the overall ultimate bearing capacity of the CFCFST stub column). The concrete reached the ultimate strength (M_2_) after the CFCFST stub column reached the ultimate bearing capacity. Therefore, the steel tube will not fail by local buckling before the CFCFST stub column reaches the ultimate bearing capacity.

**Figure 8:**
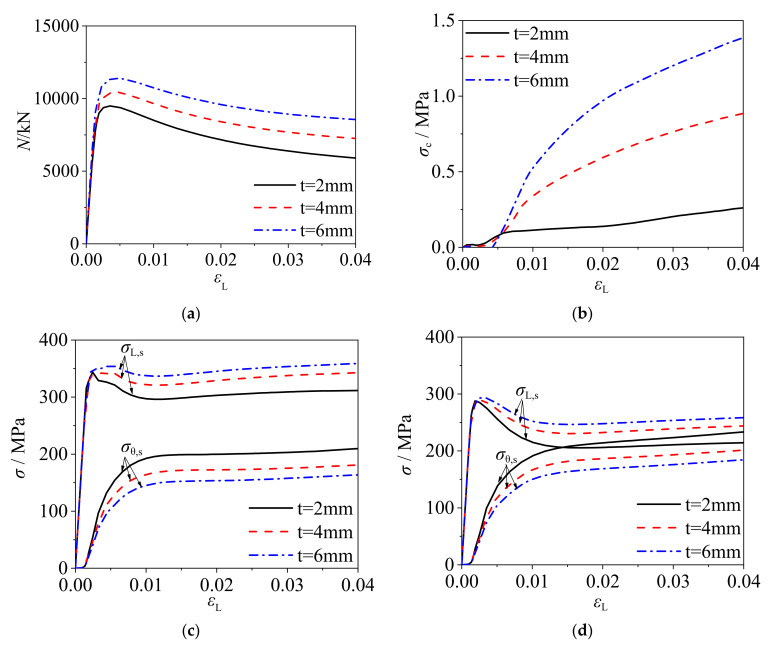
The influence of *t* on the mechanical performance of CFCFST stub columns: (**a**) load–strain curves, (**b**) radial stress–strain curves of core concrete, (**c**) stress–strain curves of steel tubes at endpoint, and (**d**) stress–strain curves of steel tubes at midpoint.

Taking the square CFCFST stub columns of *B* = 400 mm, *f*_s_ = 435 MPa, and C60 as an example, Figure 8 presents the load–strain curves, radial stress–strain curves of core concrete, and stress–strain curves of steel tube with different wall-thicknesses. It can be seen that the ultimate bearing capacity of CFCFST stub columns increased with the increase of wall-thickness (decrease of *B/t* ratio). The radial stress of core concrete increased with the decrease of *B/t* ratio and this suggests that the confinement effect of cold-formed steel tube on concrete was increased. However, as illustrated in Figure 8c,d, the decrease of axial stress and the increase of transverse stress slowed down with the decrease of *B/t* ratio, indicating that the confinement efficiency was decreased.

#### 2.3.4. Concrete Strength

**Figure 9:**
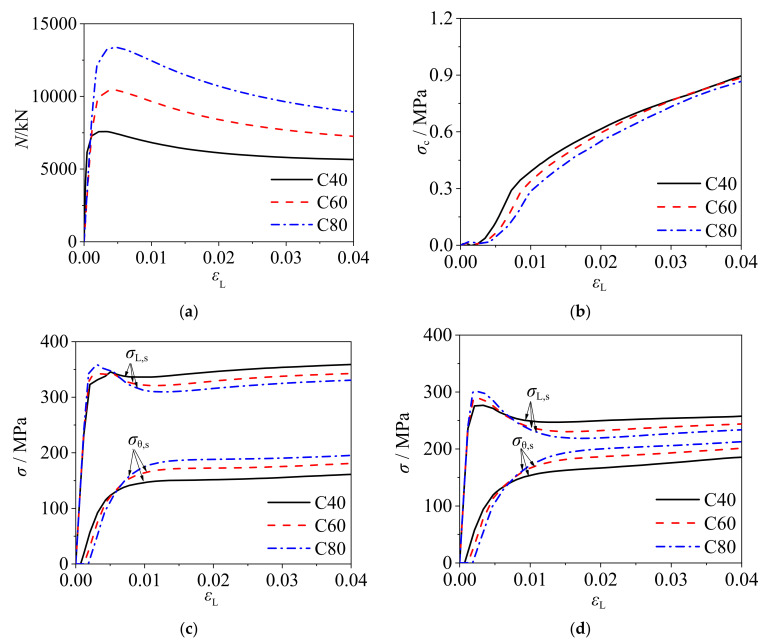
The influence of concrete strength on the mechanical performance of CFCFST stub columns: (**a**) load–strain curves, (**b**) radial stress–strain curves of concrete, (**c**) stress–strain curves of steel tubes at endpoint, and (**d**) stress–strain curves of steel tubes at midpoint.

Figure 9 presents the load–strain curves of square CFCFST stub columns, radial stress–strain curves of core concrete, and stress–strain curves of steel tube with *f*_s_ = 345 MPa and *B* × *t* × *L* = 400 mm × 4 mm × 1200 mm but different concrete strengths. It can be seen that the ultimate bearing capacity of CFCFST stub columns increased greatly with the increase of concrete strength. The radial stress of core concrete was decreased with the increase of concrete strength, as shown in Figure 9b. This reflects that the confinement effect of steel tube on core concrete is lower with higher concrete strength. However, the decreasing amplitude of axial stress and the increasing amplitude of transverse stress were both increased with the increase of concrete strength, as shown in Figure 9c. The results suggest that the confinement efficiency increases with the increase of concrete strength.

#### 2.3.5. Yield Strength of Cold-Formed Steel

**Figure 10:**
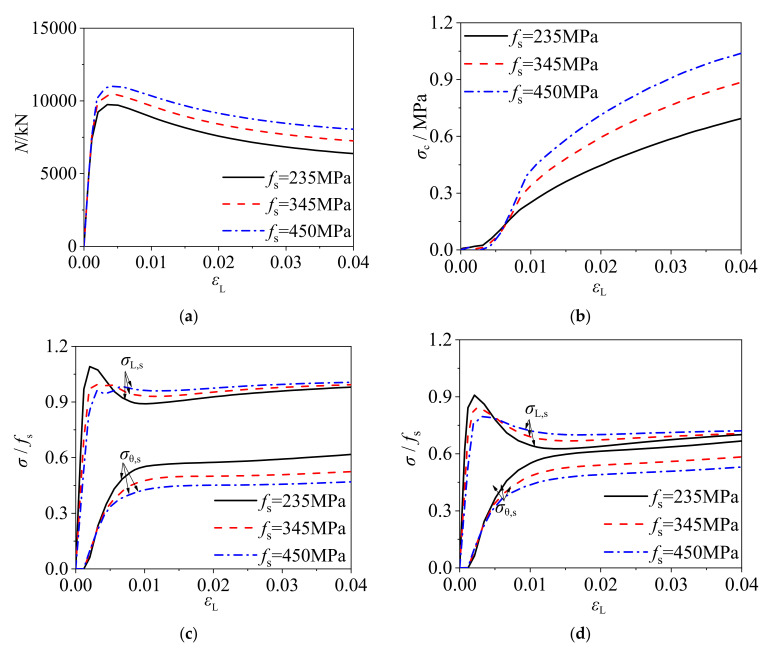
The influence of steel yield strength on the mechanical performance of CFCFST stub columns: (**a**) load–strain curves, (**b**) radial stress–strain curves of core concrete, (**c**) stress to yield strength–strain curves of steel tubes at endpoint, and (**d**) stress to yield strength-strain curves of steel tubes at midpoint.

Figure 10 shows the load–strain curves of square CFCFST stub columns, radial stress–strain curves of core concrete, and stress–strain curves of steel tube with C60 concrete and *B* × *t* × *L* = 400 mm × 4 mm × 1200 mm but different yield strengths of cold-formed steel. The ultimate bearing capacity of CFCFST stub columns and the radial stress of concrete increased with the increase of steel yield strength, as shown in Figure 10a,b. It reflects that the confinement effect of cold-formed steel tube on concrete increases with higher steel strength. However, the distance between *σ*_L,s_/*f*_s_ − *ε*_L_ curve and *σ*_θ,s_/*f*_s_ − *ε*_L_ curve increases, which indicates that the confinement efficiency decreases with the increase of steel yield strength.

## 3. Practical Design Formula for Load Bearing Capacity

### 3.1. Model Simplification

**Figure 11:**
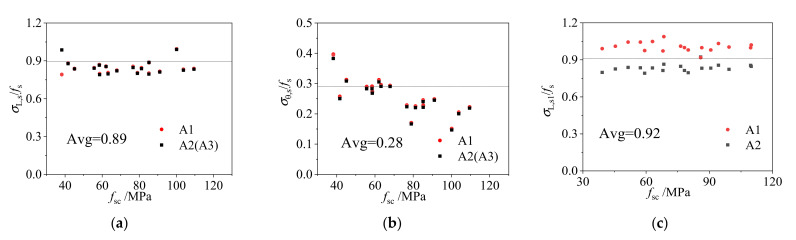

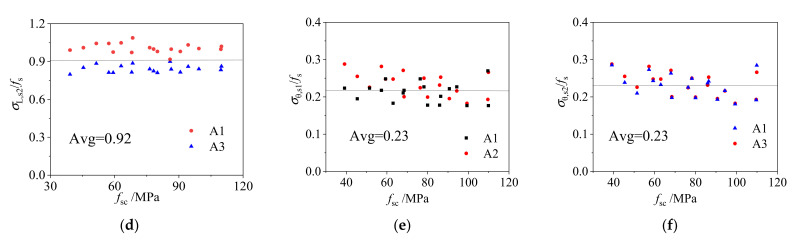
Axial stress and transverse stress of rectangular CFCFST stub columns: (**a**) average ratio of axial compressive stress to yield strength (*B/D* = 1), (**b**) average ratio of tensile transverse stress to yield strength (*B/D* = 1), (**c**) average ratio of axial compressive stress to yield strength on long-side (*B/D* = 2), (**d**) average ratio of axial compressive stress to yield strength on short-side (*B*/*D* = 2), (**e**) average ratio of tensile transverse stress to yield strength on long-side (*B*/*D* = 2), and (**f)** average ratio of tensile transverse stress to yield strength on short-side (*B*/*D* = 2).

In order to analyze the stress state of steel tube and core concrete of the CFCFST column at the ultimate state, the relation between the actual stress (axial stress *σ*_L,s_ and transverse stress *σ*_θ,s_) to the yield strength (*f*_s_) of steel tube were plotted against the overall ultimate strength (*f*_sc_ = *N*_u_/*A*_sc_, *A*_sc_ = *A*_c_ + *A*_s_) of the CFCFST column as shown in Figure 11.

Figure 11a shows the relation between *σ*_L,s_*/f*_s_ and *f*_sc_ and Figure 11b shows the relation between *σ*_θ,s_*/f*_s_ and *f*_sc_ for a square CFCFST column. Figure 11c shows the relation between *σ*_L,s_*/f*_s_ and *f*_sc_ and Figure 11e shows the relation between *σ*_θ,s_*/f*_s_ and *f*_sc_ for the long-side of a rectangular CFCFST column. Figure 11d shows the relation between *σ*_L,s_*/f*_s_ and *f*_sc_ and Figure 11f shows the relation between *σ*_θ,s_ */f*_s_ and *f*_sc_ for the short-side of a rectangular CFCFST column.

**Figure 12:**
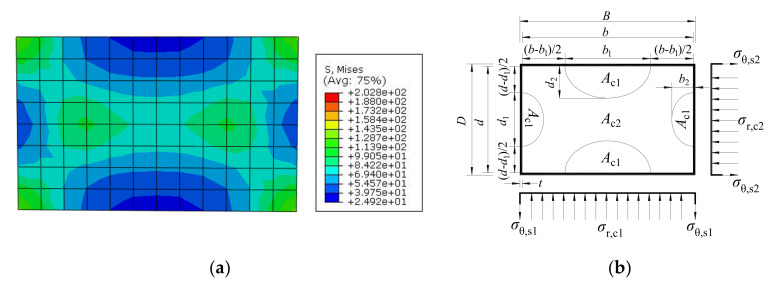
Actual FE stress nephogram and simplified stress distribution of rectangular CFCFST sections: (**a**) FE stress nephogram and (**b**) simplified stress-distribution model.

According to the stress nephogram of the core concrete at ultimate limit state in Figure 12a, the stress distribution can be simplified to different stress regions as shown in Figure 12b. It is assumed that core concrete in the unconstrained area (region of A_c1_) is not constrained by the rectangular steel tube, and the core concrete in the constrained area (region of A_c2_) is uniformly constrained by the rectangular steel tube. In Figure 12b, *b* is the long-side dimension of core concrete and *d* is the dimension of the short-side. According to the finite element analysis results, the parameters for constrained area and unconstrained area in the simplified stress-distribution model at the middle height of the CFCFST column with different *B*/*D* ratios are shown in Table 2. It can be seen that with the increase of *B*/*D* ratio of rectangular sections, the constrained area on the long-side decreased while the constrained area on the short-side increased.

The ratios of axial stress (*σ*_L,s1_) and transverse stress (*σ*_θ,s1_) on long-side and axial stress (*σ*_L,s__2_) and transverse stress (*σ*_θ,s__2_) on short-side to steel yield strength with different *B*/*D* ratios are shown in Table 3. The model simplification was completely based on the stress distribution and the superposition theory when the core concrete reaches the ultimate limit state. The ratios of *A*_c1_/*A*_c_ for the unconstrained concrete area and *A*_c2_/*A*_c_ for the constrained concrete area in Figure 12b were also calculated and shown in Table 3. It was found that with the increase of *B*/*D* ratio, the unconstrained area increased. 

### 3.2. Formulation and Validation

Validation can be obtained from the relation between the radial stress (*σ*_r,c_) of core concrete in the constrained area and the transverse stress (*σ*_θ,s_) of the steel tube in Figure 12b:

(6) $$\left\{\begin{array}{l} \sigma_{r,c1}=\frac{2\sigma_{\theta ,s1}}{B/t-2}\\ \sigma_{r,c2}=\frac{2\sigma_{\theta ,s2}}{D/t-2} \end{array}\right.$$

(7) $$A_{s1}=\alpha A_{s},A_{s2}=\beta A_{s},A_{c2}=\delta A_{c}$$

(8) $$\sigma_{\theta ,s1}=af_{s},\sigma_{\theta ,s2}=bf_{s},\sigma_{L,s1}=cf_{s},\sigma_{L,s2}=df_{s}$$

where *A*_s1_ is the sectional area of steel tube for two long-sides and *A*_s2_ is the sectional area for two short-sides.

The axial stress (*σ*_L,c_) of constrained concrete can be given as follows:

(9) $$\left\{\begin{array}{l} \sigma_{L,c}=f_{c}+k_{1}\sigma_{r,c}\\ \sigma_{r,c}=\frac{\sigma_{r,c1}+\sigma_{r,c2}}{2} \end{array}\right.$$

where *k*_1_ is the coefficient of lateral pressure and *k*_1_ = 3.4, according to Ding et al. [33].

On the basis of static equilibrium method, the ultimate bearing capacity *N*_u_ of axially-loaded CFCFST stub columns can be expressed as follows:

(10) $$N_{u}=f_{c}A_{c1}+\sigma_{L,c}A_{c2}+\sigma_{L,s1}A_{s}{}_{1}+\sigma_{L,s2}A_{s2}$$

Substituting Equations (6)–(9) into (10):

(11) $$\begin{array}{ll} N_{u} & =f_{c}A_{c1}+(f_{c}+k_{1}(\frac{t\sigma_{\theta ,s1}}{B-2t}+\frac{t\sigma_{\theta ,s2}}{D-2t}))A_{c2}+\sigma_{L,s}{}_{1}A_{s1}+\sigma_{L,s2}A_{s2}\\ & =f_{c}A_{c1}+f_{c}A_{c2}+k_{1}(\frac{t\sigma_{\theta ,s1}}{B-2t}+\frac{t\sigma_{\theta ,s2}}{D-2t}))\delta (B-2t)(D-2t)+c\alpha f_{s}A_{s}+d\beta f_{s}A_{s}\\ & =f_{c}A_{c}+k_{1}\delta (t\sigma_{\theta ,s1}(D-2t)+t\sigma_{\theta ,s2}(B-2t))+(c\alpha +d\beta )f_{s}A_{s}\\ & =f_{c}A_{c}+k_{1}\delta (Dt\sigma_{\theta ,s1}+Bt\sigma_{\theta ,s2}-2t^{2}(\sigma_{\theta ,s1}+\sigma_{\theta ,s2}))+(c\alpha +d\beta )f_{s}A_{s} \end{array}$$

The *t*^2^ is infinitesimal and negligible in Equation (11):

(12) $$\begin{array}{ll} N_{u} & =f_{c}A_{c}+k_{1}\delta (Dt\sigma_{\theta ,s1}+Bt\sigma_{\theta ,s2})+(c\alpha +d\beta )f_{s}A_{s}\\ & =f_{c}A_{c}+\frac{k_{1}\delta (A_{s2}\sigma_{\theta ,s1}+A_{s1}\sigma_{\theta ,s2})}{2}+(c\alpha +d\beta )f_{s}A_{s}\\ & =f_{c}A_{c}+(\frac{k_{1}\delta (a\beta +b\alpha )}{2}+c\alpha +d\beta )f_{s}A_{s} \end{array}$$

*N*_u_ can be rewritten as:

(13) $$N_{u}=f_{c}A_{c}+Kf_{s}A_{s}$$

In Equation (13), *K* is the confinement coefficient of rectangular CFCFST stub columns. The values of *K* can be obtained with different *B*/*D* based on the finite element analysis results, and they are summarized in Table 3. The following relation can be obtained by the fitting method:

(14) K=1.25−0.22ln(B/D)

The expression of *K* of rectangular concrete-filled mild steel tubular (CFST) stub columns with different *B*/*D* ratios suggested in reference [20] is as follows:

(15) K=1.04−0.06ln(B/D−0.93)

**Figure 13:**
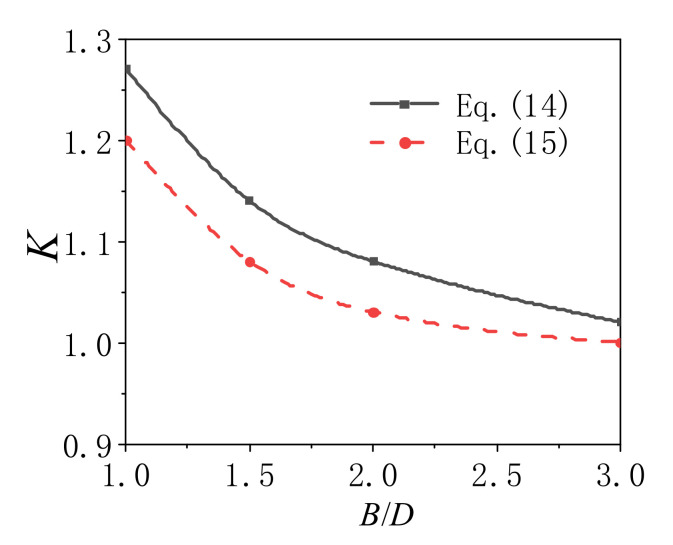
*K* of CFCFST and CFST stub columns with different *B*/*D*.

Comparisons of the values of *K* for rectangular CFCFST stub columns and CFST stub columns are shown in Table 4 and Figure 13. The confinement coefficient *K* of cold-formed square CFT stub columns was 1.25, while *K* of hot-rolled square CFT stub columns was 1.2, and that of stainless square CFT stub columns was 1.4. This indicates that the composite behavior of cold-formed steel square tube is stronger than hot-rolled steel but weaker than stainless steel.

**Figure 14:**
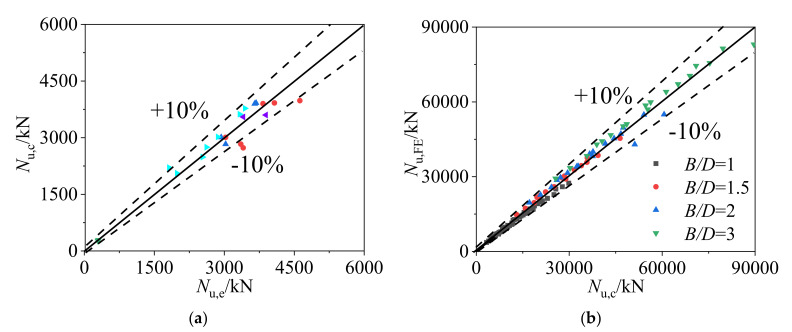
Comparisons from test and FE results versus Equation (13) results: (**a**) comparison of the ultimate bearing capacities obtained from test results and Equation (13) and (**b**) comparison of the ultimate bearing capacities obtained from FE results and Equation (13).

As shown in Table 1 and Figure 14a, the ultimate bearing capacity (*N*_u,c_) calculated by Equation (13) was compared with the experimental results (*N*_u,e_) [8,9,10,13,14,17]. The average ratio of *N*_u,e_ to *N*_u,c_ was 0.99 with the corresponding dispersion coefficient of 0.09. The calculated results were also compared with the FE results in Figure 14b, the average ratio of *N*_u,__FE_ to *N*_u,c_ was 1.02 with the corresponding dispersion coefficient of 0.06. It is demonstrated that the calculated results using Equation (13) with the obtained *K* values in this study are in good agreement with both the experimental and FE results.

## 4. Conclusions

Based on triaxial plastic-damage constitutive relations with features of the parameter certainty for the infilled concrete and a rational constitutive model for cold-formed steel, a fine finite 3D solid element model of CFCFST stub columns was established, which takes into account the composite behavior of cold-formed steel tube and core concrete. Different models of steel material have influences on the composite behavior and the four-fold line model was selected because it is closest to the stress–strain curve of cold-formed steel.

- When *B*/*t* is constant for square columns, changing the sectional length *B* has little effect on the mechanical properties of the CFCFST columns. When *B* is constant, the confinement effect increases but the confinement efficiency decreases with the increase of wall-thickness *t*. The composite behavior decreases with the increase of *B*/*D* of rectangular CFCFST columns.
- With the increase of concrete strength, the confinement effect decreases but the constraint efficiency increases. However, the results are opposite with the increase of yield strength of cold-formed steel.
- In the proposed formula, the confinement coefficient of square CFCFST stub columns in this study was 1.25, which was greater than the confinement coefficient of 1.2 for concrete-filled square hot-rolled steel tubular stub columns but smaller than the confinement coefficient of 1.4 for concrete-filled square stainless steel tubular stub columns.

## Figures and Tables

**Table 1:** Comparison of experimental results in references, FE results, and calculated results.

Ref.	Specimens	*B* × *D* × *t* (mm)	*L* (mm)	*E*_s_ (MPa)	*f*_cu_ (MPa)	*f*_s_ (MPa)	*N*_u,e_ (kN)	*N*_u,FE_ (kN)	*N*_u,c_ (kN)	*N*_u,e_/*N*_u,FE_	*N*_u,e_/*N*_u,c_
[8]	CFT-SC	150 × 100 × 3.2	450	209,000	53.6	380	1208	1224	1172	0.99	1.02
[10]	CRST1	200 × 150 × 3.4	600	206,000	39	446	2059	1988	1987	1.04	1.00
	CRST2	200 × 150 × 5.1	600	202,000	39	450	2487	2622	2536	0.95	0.93
	CRST3	200 × 150 × 5.6	600	200,000	39	410	2748	2849	2619	0.96	1.04
	CRST4	200 × 150 × 4.9	600	206,000	39	409	2207	2253	1817	0.98	1.22
	CRST5	200 × 200 × 6.1	600	192,000	26.3	406	3621	3525	3330	1.03	1.09
	CRST6	200 × 200 × 5.8	600	200,000	26.3	440	3777	3739	3443	1.01	1.10
	CRST7	200 × 200 × 4.8	600	202,000	26.3	407	3020	3655	2862	0.83	1.06
[9]	Ca6-1	200 × 200 × 6	600	206,000	20.0	393	3010	3130	2934	0.96	1.03
	Ca6-2	200 × 200 × 6	600	206,000	20.0	393	2830	3130	3029	0.90	0.93
	Ca10-1	200 × 200 × 10	600	206,000	20.0	331	3920	4060	3682	0.97	1.06
	Ca10-2	200 × 200 × 10	600	206,000	20.0	331	3900	4060	3653	0.96	1.07
[13]	Pa-6-1	197 × 197 × 6.4	600	206,000	20.5	461	2730	3066	3407	0.89	0.80
	Pa-6-2	198.5 × 198.5 × 6.1	600	206,000	20.5	406	3010	3066	3030	0.98	0.99
	Pa-6-3	200.5 × 200.5 × 6.3	600	206,000	20.5	445	2830	3066	3355	0.92	0.84
	Pa-10-1	201.0 × 201.0 × 10.3	600	206,000	20.5	424	3980	4150	4621	0.96	0.86
	Pa-10-2	201.0 × 201.0 × 10.0	600	206,000	20.5	372	3920	4150	4075	0.94	0.96
	Pa-10-3	199.5 × 199.5 × 10.1	600	206,000	20.5	348	3900	4150	3830	0.94	1.02
[14]	P1C	100 × 70 × 2.1	200	206,000	20	270	290	316	304	0.92	0.94
	P2C	100 × 70 × 2	300	206,000	20	270	270	312	294	0.87	0.91
	P3C	99 × 70 × 2	400	206,000	20	270	265	260	293	1.02	0.89
[17]	ZYB-9	300 × 200 × 5.73	1000	216,400	31	336	3550	3000	3407	1.18	1.03
	ZYB-7	300 × 200 × 5.73	800	216,400	41	336	3600	3350	3891	1.07	0.92
	Mean									0.97	0.99
	Cov									0.07	0.09

Note: *N*_u,e_ are the experimental results, *N*_u,FE_ are the FE results, and *N*_u,c_ are the calculated values by Equation (13).

**Table 2:** Parameters in the simplified stress-distribution model at the middle height of CFCFST columns with different *B*/*D*.

*B/D*	*b*_1_/*b*	*b*_2_/*b*	*d*_1_/*d*	*d*_2_/*d*
1	7/20	7/20	7/20	7/20
1.5	2/3	1/8	1/3	1/6
2	1/2	1/6	4/9	1/4
3	2/5	1/5	1/2	1/3

**Table 3:** Relationships between the axial stress, transverse stress, and yield strength of steel tubes.

*B*/*D*	σ_L,s1_/*f*_s_	σ_L,s2_/*f*_s_	σ_θ,s1_/*f*_s_	σ_θ,s2_/f_s_	*A*_c1_/*A*_c_	*A*_c2_/*A*_c_	*K*
1	0.89		0.28		0.22	0.77	1.25
1.5	0.91	0.92	0.25	0.26	0.47	0.53	1.14
2	0.92	0.92	0.23	0.23	0.59	0.41	1.08
3	0.92	0.93	0.20	0.21	0.73	0.27	1.02

**Table 4:** Comparison of *K* of CFST stub columns with different steel types.

Steel	*B/D* = 1	*B/D =* 1.5	*B/D =* 2	*B/D =* 3	Ref.
Cold-formed steel	1.25	1.14	1.08	1.02	
Hot-rolled steel	1.20	1.08	1.03	1.00	Ding et al. [20]
Stainless steel	1.40				Ding et al. [21]

## Data Availability

The data presented in this study are available on request from the corresponding author.
